# The Modulatory Role of Heme Oxygenase on Subpressor Angiotensin II-Induced Hypertension and Renal Injury

**DOI:** 10.1155/2012/392890

**Published:** 2012-03-11

**Authors:** Kiran Chandrashekar, Arnaldo Lopez-Ruiz, Ramiro Juncos, Karl Nath, David E. Stec, Trinity Vera, Ruisheng Liu, Luis A. Juncos

**Affiliations:** ^1^Division of Nephrology, Department of Medicine, University of Mississippi Medical Center, Jackson, MS 39216, USA; ^2^Department of Physiology and Biophysics, University of Mississippi Medical Center, Jackson, MS 39216, USA; ^3^Division of Nephrology, Department of Medicine, Mayo Clinic, Rochester MN, 55905, USA

## Abstract

Angiotensin II (AngII) causes hypertension (HTN) and promotes renal injury while simultaneously inducing reno-protective enzymes like heme oxygenase-1 (HO-1). We examined the modulatory role of HO on sub-pressor angiotensin II (SP-AngII) induced renal inflammation and injury. We first tested whether the SP-AngII-induced renal dysfunction, inflammation and injury are exacerbated by either preventing (chronic HO-1 inhibition) or reversing (late HO-1 inhibition) SP-AngII-induced HO (using tin protoporphyrin; SnPP). We next examined whether additional chronic or late induction of SP-AngII-induced HO (using cobalt protoporphyrin; CoPP), prevents or ameliorates renal damage. We found that neither chronic nor late SnPP altered blood pressure. Chronic SnPP worsened SP-AngII-induced renal dysfunction, inflammation, injury and fibrosis, whereas late SnPP worsened renal dysfunction but not inflammation. Chronic CoPP prevented HTN, renal dysfunction, inflammation and fibrosis, but surprisingly, not the NGAL levels (renal injury marker). Late CoPP did not significantly alter SP-AngII-induced HTN, renal inflammation or injury, but improved renal function. Thus, we conclude (a) endogenous HO may be an essential determining factor in SP-AngII induced renal inflammation, injury and fibrosis, (b) part of HO's renoprotection may be independent of blood pressure changes; and (c) further induction of HO-1 protects against renal injury, suggesting a possible therapeutic target.

## 1. Introduction

Angiotensin II (AngII) is one of the major factors playing a role in the development of chronic kidney disease. It does so by virtue of its hemodynamic, prooxidant, proinflammatory, and profibrotic effects. However, along with its detrimental effects, AngII has been found to induce adaptive, protective pathways. One such cytoprotective system is via heme oxygenase (HO). HO is the rate-limiting step in the metabolism of heme, breaking it down into biliverdin with the resultant release of ferric iron and carbon monoxide [1–3]. It exists as two isoforms, HO-1 and HO-2. HO-1 is an inducible form that is upregulated by various stimuli including lipopolysaccharide, nitric oxide, and AngII [1, 2], whereas HO-2 is constitutively expressed [3]. The cytoprotective properties of HO are attributed to the antioxidant, anti-inflammatory, and vasorelaxant actions of the end products of heme metabolism; biliverdin with the resultant bilirubin and carbon monoxide [4]. Previous studies have demonstrated the importance of HO in dampening the hypertensive and renal vasoconstrictor effects of AngII [5–7]. However, these studies employed pressor doses of AngII. Such high doses of AngII may not accurately reflect the balance between the injurious and adaptive factors that are triggered by physiological doses of AngII. A model that is more analogous to clinical situations is one in which AngII levels are elevated, but within a pathophysiologic range. This is achieved by chronically infusing subpressor doses of AngII (SP-AngII). This model is characterized by the development of salt-sensitive hypertension (HTN), increased expression of proinflammatory factors, oxidative stress, and progressive renal injury [4, 5, 8–10]. We recently examined the renal vascular effects of acute inhibition of HO after 2 weeks of SP-AngII [11]; however, the modulatory effects of HO on SP-AngII-induced renal inflammation and injury are incompletely understood. In the present study, we evaluated the role of SP-AngII-induced HO-1 in modulating the injurious effects of SP-AngII. We tested whether chronically inhibiting HO exacerbates SP-AngII-induced renal inflammation and injury and also whether inhibiting HO late in the course of SP-AngII alters the renal dysfunction. In addition, because inducing overexpression of HO-1 may represent a potential therapeutic target for preventing AngII-induced damage, we tested whether chronically inducing HO-1 prevented renal injury and whether inducing HO-1 late in the course of SP-AngII-induced HTN ameliorates renal inflammation and damage.

## 2. Methods

All experiments were approved by the Institutional Animal Care and Use Committee of the University of Mississippi Medical Center and were performed in accordance with the *Guide for the Care and Use of Laboratory Animals *of the National Institutes of Health.

### 2.1. Experimental Groups and Design

Male Sprague Dawley rats (Harlan Teklad, Indianapolis, IN USA) (263 ± 20 g) maintained on standard rat chow with water ad libitum were used in all experiments. All animals were inserted with osmotic minipumps (2ML2, Alzet minipumps, DURECT Corp., Cupertino, CA, USA), which infused either a vehicle (saline) or AngII (200 ng/kg/min) subcutaneously for 14 days as previously described [6, 12, 13]. We used this dose of SP-AngII because it increases plasma AngII to levels that do not cause immediate vasoconstriction, yet reliably cause HTN and lead to renal inflammation and injury within the time frame of the experiments. We then tested the involvement and effects of HO in these animals. For this we modulated HO activity in two ways: the first by preemptively modulating the activity of HO (treating throughout the SP-AngII infusion) and second by reversing or enhancing its activity after SP-AngII has been present for 12 days. Initially, we tested whether chronically inhibiting HO (tin protoporphyrin (SnPP) 30 *μ*mol/kg, i.p., every 3 days) worsens renal inflammation and injury. We then tested whether blocking the effect of HO near the end of the 2 -week infusion (late SnPP; 50 *μ*mol/kg, i.p. on day 12), worsens the renal parameters. Because further induction of HO-1 may protect against AngII-induced HTN, we tested whether chronic induction of HO-1 (cobalt protoporphyrin (CoPP) 30 *μ*mol/kg, i.p., every 3 days) blunts renal inflammation and injury. Finally, we tested whether inducing HO-1 near the end of the SP-AngII infusion (late CoPP; 50 *μ*mol/kg, i.p, on day 12) can ameliorate the adverse affects of SP-AngII. 

 Systolic blood pressure (SBP) was measured during the 14-day course of SP-AngII/vehicle infusion by tail cuff plethysmography (TCP) (Harvard Apparatus, Holliston MA, USA) in conscious, trained animals. On day 14 of the protocol, blood was collected and the animals were housed in metabolic cages, and urine was collected for 24 hours. The animals were then sacrificed, and the kidneys were harvested. All organs were weighed and flash-frozen using liquid nitrogen. 

### 2.2. Measurements

#### 2.2.1. Renal Function, Oxidative Stress, Inflammation, and Injury

Renal function was assessed by measuring plasma creatinine using a Quantichrom Creatinine Assay kit (BioAssay Systems). The kidney cortices were homogenized using a tissue homogenizer (IKA Works) in radioimmunoprecipitation assay (RIPA) buffer (10 *μ*L of phenylmethylsulfonyl fluoride (PMSF) +10 *μ*L sodium orthovanadate +10–20 *μ*L protease inhibitor cocktail per mL of 1X RIPA lysis buffer). A bicinchoninic acid (BCA; Pierce, Rockford, IL, USA) protein assay kit was used for the calorimetric detection and quantification of the total protein in the kidney homogenates. Renal inflammation was estimated by analyzing the renal interleukin-6 levels (IL-6). Kidney cortex homogenates from all groups were analyzed simultaneously for IL-6 levels (Quantikine Rat IL-6 Immunoassay kit) (R&D Systems, Minneapolis MN, USA). Finally renal injury was determined by measuring the following: urinary levels of neutrophil gelatinase-associated lipocalin levels (NGAL, by ELISA; Assay Designs, Ann Arbor, MI, USA); renal tissue levels of kidney injury marker (KIM-1, by ELISA; R&D Systems, Minneapolis, MN, USA); apoptosis, as measured by cytochrome-C levels (by ELISA; R&D Systems, Minneapolis, MN, USA), and the profibrotic signal, transforming growth factor- beta 1 (TGF-*β*1; by ELISA; R&D Systems, Minneapolis, MN, USA).

### 2.3. HO Activity

HO activity was determined by measuring the amount of bilirubin produced using a well established protocol [14]. Briefly, the kidney cortices were processed using a homogenization buffer (sucrose, monobasic and dibasic potassium phosphate, EDTA and PMSF at ph 7.7) The protein content of this homogenate was measured and then mixed with a potassium phosphate buffer (monobasic and dibasic potassium phosphate with water at ph 7.4), Glucose-6-phosphate, glucose-6-phosphate dehydrogenase, beta-nicotinamide diamine phosphate (*β*-NADP) and hemin to get a 1.2 mL reaction. The reaction was then incubated for 1 hr at 37°C in a dark room, after which 1.2 mL of chloroform was added and the mixture vortexed for 15 seconds. The reaction was then frozen overnight, thawed and vortexed. The samples were centrifuged at 15,000 g for 10 minutes, resulting in a ring-like layer separating the supernatant from the lower half. The supernatant was discarded and the absorbance was measured at 464 and 530 nm in a spectrophotometer, using an extinction coefficient of 40 mM/cm for bilirubin. The resulting HO activity was expressed as nmoles of bilirubin/mg of kidney protein/hr (nmol bil/mg protein/hour). 

### 2.4. Statistics

All variables were expressed as mean ± SEM. An unpaired Student's *t*-test was used to perform comparisons between two groups. The ANOVA test was employed to statistically compare two or more groups. Statistical significance was established at a *P* value less than 0.05. We utilized the Instat software (GraphPad, v3.06; La Jolla, CA, USA) to analyze all data.

## 3. Results

### 3.1. HO Activity (Figure 1)

We first examined whether our therapeutic protocols were effective in inhibiting or inducing HO activity. SP-AngII increased HO activity from 0.9 ± 0.01 to 2.1 ± 0.04 nmoles of bilirubin/mg protein/hour. Neither chronic nor late administration of SnPP decreased HO activity in control animals (0.63 ± 0.33 and 0.8 ± 0.01 nmoles of bilirubin/mg protein/hour, resp.) but both lowered HO activity in SP-AngII treated rats (from 2.1 ± 0.04 to 0.7 ± 0.02 and 0.7 ± 0.12 nmoles of bilirubin/mg protein/hour, resp.). Chronic and late induction of HO-1 with CoPP increased HO activity in control animals (from 0.9 ± 0.01 to 2.1 ± 0.3 and 1.3 ± 0.2 nmoles of bilirubin/mg protein/hour, resp.) and further elevated SP-AngII-induced HO activity (from 2.1 ± 0.04 to  3 ± 0.25  and  3 ± 0.21 nmoles of bilirubin/mg protein/hour, resp.).

### 3.2. Blood Pressure (Figure 2)

Chronic infusion of Sp-Ang II increased the blood pressure from 122 ± 1 mm Hg to 167 ± 2 mm of Hg. Neither chronic nor late inhibition of HO augmented SP-AngII- induced HTN (167 ± 2 versus  174 ± 2  and  167 ± 2  versus 173 ± 1, resp.). On the other hand, chronic induction of HO-1 prevented the SP-AngII-induced elevation in SBP (SBP was 123 ± 4 mmHg after 2 weeks of SP-AngII). Late induction did not significantly blunt SP-AngII-induced HTN (154 ± 10 mmHg). Neither inhibition nor induction of HO altered blood pressure in vehicle-treated animals.

### 3.3. Renal Function, Inflammation and Injury

**Figure 3:**
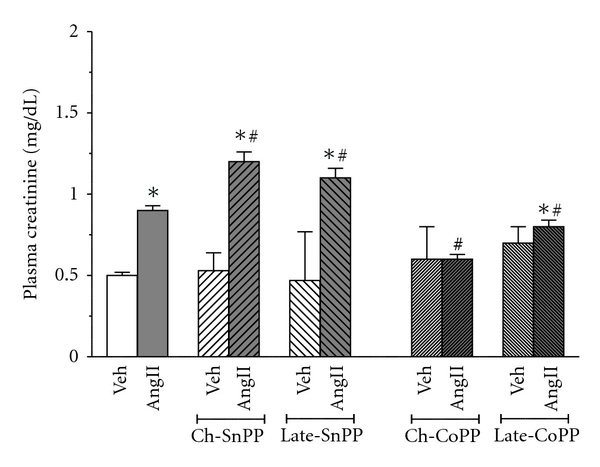
Effect of SP-Ang II and HO modulation on the plasma creatinine levels was determined in the following groups; Vehicle (Veh) treated (*n* = 6), SP-AngII-treated (*n* = 6), chronic-SnPP- (Ch-SnPP-) treated (*n* = 6), SP-AngII/Ch-SnPP-treated (*n* = 6), late SnPP-treated (*n* = 5), SP-AngII/late-SnPP-treated (*n* = 5), chronic-CoPP- (Ch-CoPP-) treated (*n* = 6), SP-AngII/Ch-CoPP–treated (*n* = 6), and late-CoPP-treated (*n* = 5), SP-AngII/late CoPP-treated (*n* = 6) rats. *P < 0.05 versus. control, ^#^ *P* < 0.05 versus. SP-AngII.

 SP-AngII increased plasma creatinine from 0.5 ± 0.02 mg/dL to 0.9 ± 0.03 mg/dL, suggesting that this dose of SP-AngII impairs renal function (Figure 3). Chronic inhibition of HO exacerbated SP-AngII-induced increases in plasma creatinine (1.2 ± 0.06 mg/dL). Late HO inhibition, despite having only a couple of days to exert its effect, also elevated SP-AngII-induced increases in plasma creatinine (1.1 ± 0.06 mg/dL). As with blood pressure, chronic induction of HO-1 prevented the rise in plasma creatinine caused by SP-AngII (0.6 ± 0.03 mg/dL), whereas late induction of HO-1 blunted this increase (0.8 ± 0.04 mg/dL). Neither inhibition nor induction of HO altered plasma creatinine in vehicle-treated animals.

**Figure 4:**
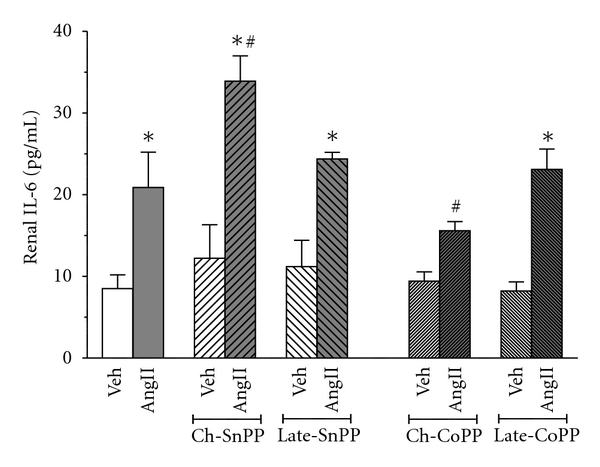
Effect of SP-Ang II and HO modulation on the inflammatory cytokine- IL-6 was estimated in the following groups; Vehicle (Veh) treated (*n* = 6), SP-AngII-treated (*n* = 6), chronic-SnPP- (Ch-SnPP-) treated (*n* = 6), SP-AngII/Ch-SnPP-treated (*n* = 6), late-SnPP-treated (*n* = 5), SP-AngII/late-SnPP-treated (*n* = 5), chronic-CoPP- (Ch-CoPP-) treated (*n* = 6), SP-AngII/Ch-CoPP–treated (*n* = 6), late-CoPP-treated (*n* = 5), SP-AngII/late CoPP-treated (*n* = 6) rats. *P < 0.05 versus. control, ^#^ *P* < 0.05 versus. SP-AngII.

Renal inflammation was assessed by measuring IL-6 levels (Figure 4). SP-AngII increased IL-6 levels from 8.5 ± 1.7 pg/*μ*g/mL to 20.9 ± 4.3 pg/*μ*g/mL. Chronic HO inhibition accentuated SP-AngII-induced IL-6 levels (to 33.9 ± 3.1 pg/*μ*g/mL). In contrast to plasma creatinine, late HO inhibition did not exacerbate the SP-AngII-induced IL-6 (24.4 ± 0.8 pg/*μ*g/mL). Chronic induction of HO-1 did not completely block SP-AngII-induced increases in IL-6 but significantly blunted it (15.6 ± 1.1 pg/*μ*g/mL), whereas late induction did not significantly alter IL-6 levels (23.1 ± 2.5 pg/*μ*g/mL). Neither inhibition nor induction of HO altered IL-6 in vehicle-treated animals.

**Figure 5:**
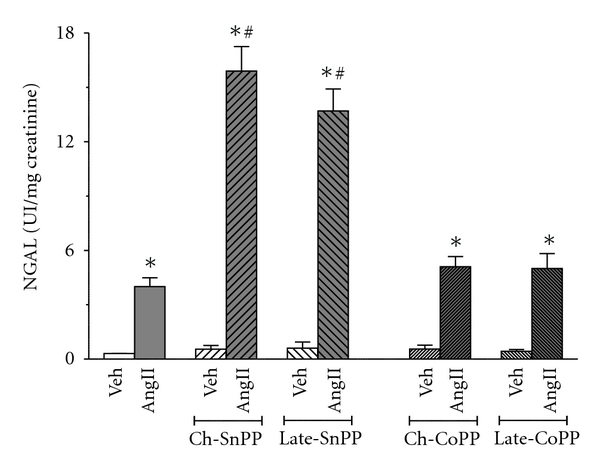
Effect of SP-Ang II and HO modulation on Urinary NGAL excretion was measured in the following groups; Vehicle- (Veh-) treated (*n* = 6), SP-AngII-treated (*n* = 6), chronic-SnPP- (Ch-SnPP-) treated (*n* = 6), SP-AngII/Ch-SnPP-treated (*n* = 6), late-SnPP-treated (*n* = 5), SP-AngII/late SnPP-treated (*n* = 5), chronic-CoPP- (Ch-CoPP-) treated (*n* = 6), SP-AngII/Ch-CoPP–treated (*n* = 6), late-CoPP-treated (*n* = 5), SP-AngII/Late-CoPP-treated (*n* = 6) rats. *P < 0.05 versus. control, ^#^ *P* < 0.05 versus. SP-AngII.

**Figure 6:**
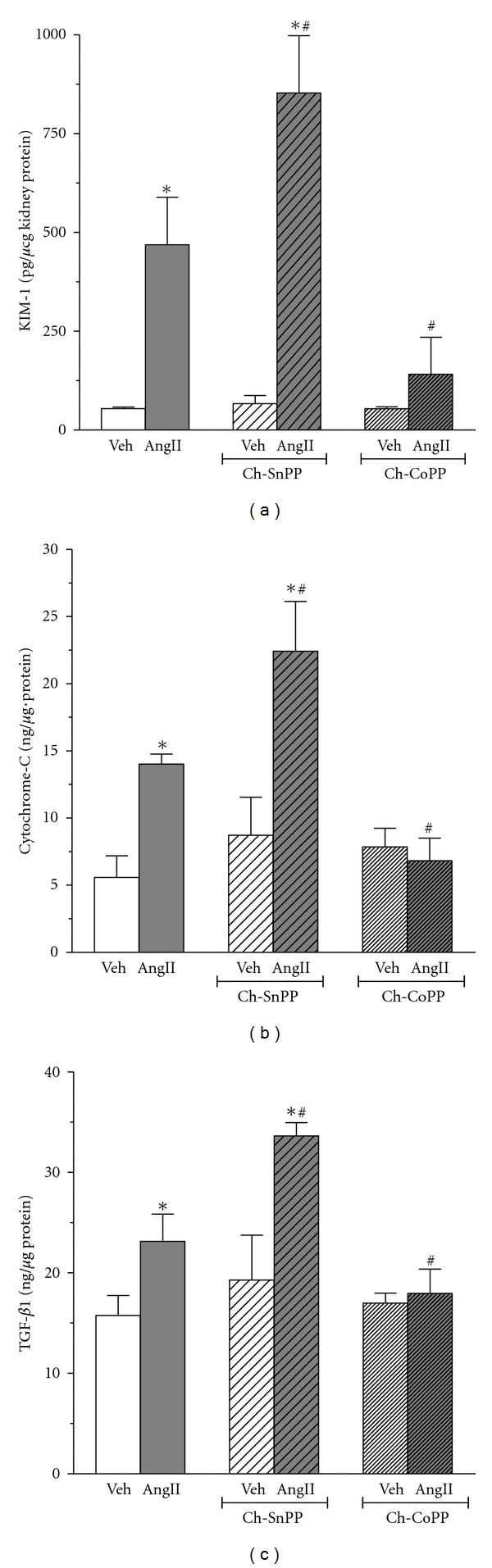
Effect of SP-Ang II and HO modulation on renal (a) KIM-1, (b) cytochrome c, and (c) TGF-*β*1 expression was estimated in the following groups; Vehicle (Veh) treated (*n* = 6), SP-AngII-treated (*n* = 6), Chronic SnPP (Ch-SnPP)-treated (*n* = 6), SP-AngII/Ch-SnPP-treated (*n* = 6), Chronic CoPP (Ch-CoPP)-treated (*n* = 6), SP-AngII/Ch-CoPP-treated (*n* = 6), *p < 0.05 versus. Control, ^#^ *p* < 0.05 versus. SP-AngII.

Renal damage was assessed by measuring the levels of the renal injury marker NGAL (Figure 5). SP-AngII increased NGAL from 0.3 ± 0.01 to 4 ± 0.49 UI/mg creatinine, thus suggesting that this dose of Ang II was inducing renal damage. Chronic HO inhibition markedly exacerbated SP-AngII-induced NGAL (to 15.9 ± 1.35 UI/mg creatinine). Late HO inhibition, despite having only a short duration to act, also increased SP-AngII-induced NGAL (to 13.7 ± 1.21 UI/mg creatinine). However, neither chronic nor late induction of HO-1 blunted the SP-AngII-induced NGAL elevation (5.1 ± 0.56 and 5 ± 0.82 UI/mg creatinine, resp.), which is in marked contrast to their effects on blood pressure, renal function and inflammation. This raises the possibility that SP-AngII-induced NGAL might not be always in tandem with co-existing renal injury. Hence we assessed additional markers of renal injury such as KIM-1 (biomarker of injury), cytochrome C (apoptosis) and TGF-*β*1(fibrosis). Since we were mainly interested in determining whether inducing HO-1 blunts frank injury, we only measured these parameters in the animals with chronic HO inhibition and induction. As shown in Figure 6(a), SP-AngII increased KIM-1 (from 54.5 ± 3.7 to 468.9 ± 120.6 pg/*μ*g kidney protein) was increased by chronic HO inhibition (853.3 ± 145.1 pg/*μ*g kidney protein), and blunted by chronic induction (140.8 ± 92.55 pg/*μ*g kidney protein). This same pattern was seen with both cytochrome c and TGF-*β*1, except that SP-AngII induced increases in these parameters was completely blocked by chronic CoPP, (Figures 6(b) and 6(c), resp.). 

## 4. Discussion

HO-1 is an important cytoprotectant which is induced in the kidney by oxidative stress, injury and certain hormones including AngII, and thus its role in modulating renal injury has been increasingly studied. Our laboratory has a longstanding interest in the interactions between AngII and HO in determining renal function and injury. In our recent study, we examined the effect of acutely inhibiting HO on renal hemodynamics in rats that were treated with very low doses of SP-AngII (50 ng/kg/min IV) [11]. We found that although renal HO-1 was not increased by this dose of SP-AngII, blocking it still modulated renal hemodynamics in SP-AngII-treated but not control rats. The present study is an extension of our previous one in that we now evaluated the impact of modulating HO activity on SP-AngII-induced renal inflammation and injury. We modulated HO activity by preemptively/chronically modulating the activity of HO, and by reversing or enhancing its activity late in the course of SP-AngII-induced HTN. We found that chronic and late HO inhibition exacerbated SP-AngII-induced renal dysfunction without significantly increasing the blood pressure. We also found that chronic induction prevented SP-AngII-induced HTN as well as the majority of the parameters of renal inflammation and injury, whereas late-induction did not significantly reduce blood pressure, renal inflammation or NGAL, but improved plasma creatinine.

AngII is an important contributor to the pathogenesis of various forms of HTN and is also a major determinant in the progression of chronic kidney disease by virtue of its hemodynamic, proinflammatory, and profibrotic effects [15–17]. Indeed, numerous studies have demonstrated that the chronic infusion of AngII causes HTN, decreases GFR, while simultaneously inducing inflammatory signaling leading to renal injury, apoptosis and fibrosis [1, 2, 18]. However, most of these studies used very high pressor doses of AngII which induce rapid and aggressive renal damage, regardless of the presence of any cytoprotection, and hence may not reflect the sequence of inflammation and injury in human disease. Our interests lie in studying the balance between the injurious and cytoprotective factors that are induced by more clinically relevant levels of AngII. In previous studies, we used doses of SP-AngII that increase oxidative stress and proinflammatory signaling, but do not consistently cause sustained HTN nor cause frank renal injury. In the present study our aim was to evaluate the modulatory effect of HO on SP-AngII-induced renal inflammation and injury. Thus we modified our SP-AngII model slightly to one that consistently increases blood pressure (it increased within 4 days) and leads to renal injury within the 2-week timeframe of our experiments, and HO-1 induction. Therefore, this model of SP-AngII is a valuable tool to examine the modulatory effects of HO on renal injury.

Various previous studies have found discrepant effects of HO inhibition on HTN [11, 14, 19]. Several studies have found that blocking HO results in an increase in blood pressure, whereas others show no change or even a decrease [11, 19]. Indeed, we previously found that HO blockade decreased mean arterial pressure (MAP) in anesthetized SP-AngII rats [11], which appeared to be due to a fall in cardiac output. The differences in blood pressure responses in the various studies may be due to differences in the species and protocols of AngII used. In the present study, we found that SnPP did not alter SBP in conscious SP-AngII rats, which at first glance appears to contradict our previous study. However, this variation may be due to the different SP-AngII models used, or to how blood pressure was measured. Indeed, the SnPP rats in the current study also tended to have a lower MAP when measured under anesthesia (data not shown).

Despite not altering the blood pressure, SnPP aggravated all the renal injury parameters suggesting that SP-AngII-induced HO is markedly attenuating the deleterious actions of AngII on the kidney. Interestingly, even late inhibition of HO activity caused a deleterious effect on renal function and NGAL levels. While, the mechanism by which late HO inhibition worsened renal function is likely due to the hemodynamic effects of HO inhibition [11], the mechanisms for the increased NGAL levels are unclear. Because NGAL does not represent cumulative injury, but rather ongoing and progressive renal injury [20], its increase may denote an accentuation in the rate of renal injury following late administration of SnPP that may be due to HO-inhibition or a direct renal toxic effect of SnPP. We speculate that the increase in NGAL was not emulated by changes in IL-6 because the relatively brief duration of HO inhibition was insufficient to detect worsening of renal inflammation. Altogether, our findings suggest that SP-AngII-induced HO tempers renal injury in a manner that is not reliant on lowering blood pressure.

Because HO-1 has profound renoprotective effects in a variety of renal diseases, there has been much interest in examining the therapeutic potential of inducing the HO-1 system in an attempt to ameliorate progression of renal injury. We therefore examined whether further induction of HO-1 during SP-AngII can further protect against SP-AngII-induced renal injury. We found that chronically inducing HO-1 completely blocked SP-AngII-induced HTN and real dysfunction. It also blunted the increase in renal inflammation, but surprisingly not the NGAL levels. These results were puzzling to us and therefore we further evaluated renal injury with other parameters; KIM-1, cytochrome c, and TGF-*β*1. We found that chronic HO-1 induction completely blocked SP-AngII-induced increases in all 3 parameters suggesting prevention of renal injury despite the high NGAL level. Although the reason for this discrepancy is unclear, it insinuates that under certain conditions NGAL may be induced independent of injury. However, we cannot discard the possibility that NGAL may simply be the earliest of the markers we measured to increase with injury.

In contrast to chronic induction, late HO-1 induction did not normalize blood pressure, nor did it alter IL-6 or NGAL. This was not unexpected as it seems unlikely that the brief duration of additional HO-1 induction would be sufficient to reverse the 12 days of SP-AngII-induced renal inflammation and injury. However, despite the lack of effect on blood pressure, late HO-1 induction significantly improved renal function, providing further evidence that under these conditions HO modulates renal but not systemic hemodynamics.

In summary, our results show that Sp-AngII-induced HO blunts the deleterious effects of SP-AngII on renal hemodynamics, inflammation and injury without significantly lowering blood pressure. Moreover, if induced early enough, HO may prevent SP-AngII-induced HTN and renal injury. Hence, we speculate that the activity of endogenous HO is an important determinant in the progression of SP-AngII-induced HTN and renal injury, and that at least part of its renoprotective effect is independent of its blood pressure lowering properties. Thus, conditions which lower HO activity may render the kidney more susceptible to injury [12, 13], while also raising the possibility of targeting HO-1 induction as a therapeutic measure to protect against renal injury.

## Figures and Tables

**Figure 1:**
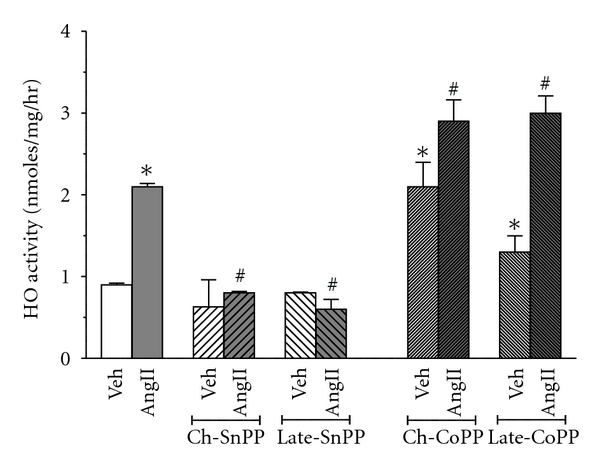
HO activity after chronic and late HO modulation. HO activity is determined by measuring the bilirubin formation in the following groups. Vehicle- (Veh-) treated (*n* = 6), SP-AngII-treated (*n* = 6), chronic-SnPP- (Ch-SnPP-) treated (*n* = 6), SP-AngII/Ch-SnPP-treated (*n* = 6), late-SnPP-treated (*n* = 5), SP-AngII/late-SnPP-treated (*n* = 5), chronic-CoPP- (Ch-CoPP-) treated (*n* = 6), SP-AngII/Ch-CoPP-treated (*n* = 6), late-CoPP-treated (*n* = 5), SP-AngII/late-CoPP-treated (*n* = 6) rats. *P < 0.05 versus. control, ^#^ *P* < 0.05 versus. SP-AngII.

**Figure 2:**
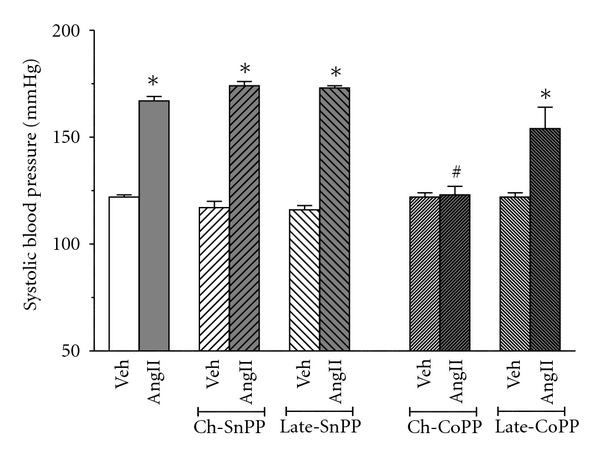
Effect of SP-Ang II and HO modulation on the SBP in the following groups Vehicle- (Veh-) treated (*n* = 6), SP-AngII-treated (*n* = 6), chronic-SnPP- (Ch-SnPP-) treated (*n* = 6), SP-AngII/Ch-SnPP-treated (*n* = 6), late-SnPP-treated (*n* = 5), SP-AngII/late-SnPP-treated (*n* = 5), chronic-CoPP- (Ch-CoPP-) treated (*n* = 6), SP-AngII/Ch-CoPP–treated (*n* = 6), Late CoPP-treated (*n* = 5), SP-AngII/late-CoPP-treated (*n* = 6) rats. *P < 0.05 versus. control, ^#^ *P* < 0.05 versus. SP-AngII.
